# Association study of morpho-phenological traits in quinoa (*Chenopodium quinoa* Willd.) using SSR markers

**DOI:** 10.1038/s41598-024-56587-0

**Published:** 2024-03-12

**Authors:** Ebrahim Souri Laki, Babak Rabiei, Hassan Marashi, Vahid Jokarfard, Andreas Börner

**Affiliations:** 1 Department of Plant Production and Genetic Engineering, Faculty of Agricultural Sciences, University of Guilan, PO Box: 41635-1314, Rasht, Iran; 2 Department of Biotechnology and Plant Breeding, Faculty of Agriculture, University of Ferdowsi, Mashhad, Iran; 3 Department of Gene Bank, Institute of Plant Genetics and Crop Plant Research, Corrensstr. 3, Seeland/OT, Gatersleben Germany

**Keywords:** Association study, General linear model, Grain yield and yield components, Marker-trait association, Multiple linear model, Yield components, Plant breeding, Plant genetics

## Abstract

In this study, the genetic and molecular diversity of 60 quinoa accessions was assessed using agronomically important traits related to grain yield as well as microsatellite (SSR) markers, and informative markers linked to the studied traits were identified using association study. The results showed that most of the studied traits had a relatively high diversity, but grain saponin and protein content showed the highest diversity. High diversity was also observed in all SSR markers, but KAAT023, KAAT027, KAAT036, and KCAA014 showed the highest values for most of the diversity indices and can be introduced as the informative markers to assess genetic diversity in quinoa. Population structure analysis showed that the studied population probably includes two subclusters, so that out of 60 quinoa accessions, 29 (48%) and 23 (38%) accessions were assigned to the first and second subclusters, respectively, and eight (13%) accessions were considered as the mixed genotypes. The study of the population structure using Structure software showed two possible subgroups (K = 2) in the studied population and the results of the bar plot confirmed it. Association study using the general linear model (GLM) and mixed linear model (MLM) identified the number of 35 and 32 significant marker-trait associations (MTAs) for the first year (2019) and 37 and 35 significant MTAs for the second year (2020), respectively. Among the significant MTAs identified for different traits, the highest number of significant MTAs were obtained for grain yield and 1000-grain weight with six and five MTAs, respectively.

## Introduction

Quinoa (*Chenopodium quinoa* Willd.) is an allotetraploid (2n = 4x = 36) that shows amphidiploid inheritance for most qualitative traits^1^. It is one of the most important crops in the South American regions^2^. It is also compatible with desert conditions, warm and dry, frosty and cold climates and can grow in areas with 40–88% moisture and survives − 4 to − 38 °C temperature^3–6^.

Iran is a vast country with varied climatic conditions in which a large part of it is dry. Soil in most parts of Iran has limitations in terms of texture and physical and chemical properties^7^. The central desert of Iran is prone to farming, but no plant can grow due to water deficit. In such conditions, quinoa cultivation can be developed due to low water requirement and contribute to employment in these areas. Since quinoa is rich in protein, it is an outstanding alternative to rice and can even be used in mixed with rice.

Yield and yield-related traits are the most important traits which are controlled by the quantitative trait loci (QTLs), and influenced by the environment^8^. Breeding yield through conventional methods has slow results due to poor understanding of the genetic control of yield. Molecular markers especially simple sequence repeats or microsatellites (SSRs) are helpful tools for identifying the genomic regions associated with yield and its related traits and can facilitate the conventional breeding programs using indirect selection by marker-assisted selection (MAS) method^9^. Microsatellites are one of the greatest achievements of biologists among all kinds of molecular markers in identifying genetic diversity inter- and intra-species. Codominant inheritance, extension on the genome, high level of polymorphism, reproducibility, and simplicity make them effective and useful for many applications^10^. Previous studies have shown that microsatellites are highly polymorphic due to the variability in the number of sequence repeats and therefore have been used as important markers in genetic mapping and population studies in many organisms^11^. Recently, QTL mapping has been used to identify the gene loci involved in the morphological and physiological traits as well as to explore their inheritance and the nature of gene action^12^. In the last decade, the application of molecular markers along with accurate phenotyping has led to the identification of several QTLs for improving grain yield^13^.

Linkage mapping and association study are among the important quantitative genetic methods that have been used in order to know the relationships between a genotype and a specific phenotype. Although the success of linkage mapping in identifying the QTLs has been proven, given that the detected QTL is more than a few centimorgans and contains hundreds of genes, identifying the suitable candidate QTL is dificult^14,15^. Several generations of selfing to provide the mapping populations such as recombinant inbred lines through controlled crosses is another limitation of the linkage mapping^16^. In contrast, association study as an alternative method to identify the relationship between a marker and a trait has many advantages compared to QTL mapping, including the use of natural populations, increasing QTL resolution and increasing allelic coverage^17^. This method is more compatible with germplasm and genetic diversity, and it allows the mapping of several traits simultaneously. Therefore, there is no need to create two-parental populations for each trait which results in extra costs for genotypic and phenotypic evaluation. It is also widely used in human and animal genetics, where creating a large segregated population is impossible^18^.

The first genetic linkage map in quinoa was constructed based on 230 AFLP, 19 SSR, and 6 RAPD markers^11^. This genetic map consisted of 35 linkage groups with a total length of 1020 cM and an average distance of 4 cM between markers. Their results showed that SSR markers indicated higher heterozygosity than AFLP and RAPD markers, which can be used in genetic mapping and quinoa breeding programs^11^. Jarvis et al.^19^ used 216 SSR markers with an average heterozygosity of 57.0 to construct the linkage map of quinoa. Their map included 38 linkage groups with a total length of 913 cM^19^. Rodriguez and Isla^20^ evaluated and compared the genetic diversity of quinoa genotypes using AFLP markers and 20 morphological traits. They prepared a similarity tree diagram based on AFLP markers and showed that it is possible to identify molecular similarities and differences between quinoa genotypes that might be associated with important morphological traits such as grain color, panicle color, phenology and geographic distribution^20^. El-Harty et al.^21^ also evaluated the genetic diversity of 32 quinoa genotypes based on 17 qualitative and 11 quantitative traits under the Saudi Arabia conditions and separated the studied genotypes into four different major groups. They also showed that the genotypes from the South American region had higher genetic diversity compared to the other studied regions based on various indices, including the average number of alleles, Shannon’s index, Nei’s genetic diversity and polymorphic percentage^21^.

Although the association study method has been used to identify desirable alleles for different traits in different plants^15,22–24^, there are relatively few reports on quinoa. In this study, a diverse set of quinoa germplasm was assessed using some important characteristics related to yield and yield components as well as microsatellite markers. The objectives of the current study were to evaluate the genetic diversity in germplasm based on morphological traits and molecular markers and to identify informative markers associated with yield and yield components by association study.

## Materials and methods

### Plant materials and phenotypic assessment

A collection of 60 quinoa accessions was used in this study. All genotypes were obtained from the IPK Gene Bank, Leibniz Institute of Plant Genetics and Crop Plant Research, Germany. The origin and the accession number of the studied genotypes are presented in Table 1. The phenotypic evaluations were carried out at the experimental fields in Kuhdasht (47° 36′ E, 33° 32′ N, and altitude of 1195 m) and Poldokhtar (47° 42′ E, 33° 09′ N, and altitude of 673 m) counties, Lorestan province, Iran, in two cropping years, 2019–2020 and 2020–2021. To perform the experiments, a randomized complete block design with three replications was used. Seeds of each genotype were planted in three rows of 10 m at a depth of 2 cm with a distance of 40 cm between rows and 3–5 cm between plants on the rows. To measure the studied traits, a random sample consisting of 15 plants was used from each plot by removing the marginal effect. The measured traits included number of days to flowering, days to physiological maturity, plant height (cm), panicle length (cm), grain length(mm), grain saponin (%), grain protein (%), 1000-grain weight (g), grain yield (kg ha^−1^), and harvest index.

**Table 1:** Accession number, origin and seed color of the studied quinoa genotypes.

Row	Genotype	Cod	Source	Seed color	No.	Genotype	Cod	Source	Seed color
1	CHEN67	D2190	Peru	Brown	31	CHEN196	D9407	Chile	Yellow
2	CHEN68	D2191	Peru	Golden-brown	32	CHEN199	D9409	Peru	Bright-white
3	CHEN71	D2196	Chile	Light brown	33	CHEN202	D9413	Peru	White
4	CHEN83	D2194	Bolivia	Bright-white	34	CHEN301	D9789	Chile	White
5	CHEN84	D2195	Bolivia	White	35	CHEN204	D9415	Chile	Golden
6	CHEN89	D5078	Bolivia	Bright	36	CHEN205	D9416	Chile	White
7	CHEN90	D5079	Chile	White	37	CHEN206	D9417	Chile	Golden
8	CHEN91	D5081	Bolivia	Golden	38	CHEN207	D9418	Chile	Bright
9	CHEN115	D9316	Bolivia	White	39	CHEN209	D9420	Chile	Bright-white
10	CHEN119	D9319	Bolivia	Whitish-yellow	40	CHEN210	D9421	Chile	White
11	CHEN121	D9336	Chile	Yellow	41	CHEN212	D9426	Chile	Golden
12	CHEN123	D9428	Peru	White	42	CHEN214	D9429	Peru	White
13	CHEN126	D9339	Peru	Bright	43	CHEN215	D9730	Peru	Bright
14	CHEN128	D9320	Chile	Whitish-yellow	44	CHEN216	D9431	Peru	White
15	CHEN133	D9361	Bolivia	Yellow	45	CHEN217	D9432	Chile	Bright
16	CHEN146	D9374	Bolivia	Bright-white	46	CHEN218	D9434	Chile	Whitish-yellow
17	CHEN151	D9382	Chile	White	47	CHEN220	D9439	Peru	Yellow
18	CHEN154	D9385	Peru	White	48	CHEN223	D9442	Chile	Bright-white
19	CHEN156	D9390	Chile	Golden	49	CHEN225	D9443	Peru	White
20	CHEN159	D9376	Bolivia	Brown	50	CHEN255	D9502	Chile	White
21	CHEN167	D9346	Chile	Yellow	51	CHEN268	D9548	Chile	Bright-white
22	CHEN171	D9350	Chile	Bright-white	52	CHEN270	D9558	Chile	Yellow
23	CHEN172	D9351	Peru	White	53	CHEN297	D9786	Chile	Bright-white
24	CHEN179	D9358	Chile	White	54	CHEN299	D9788	Chile	White
25	CHEN182	D9392	Peru	White	55	CHEN328	D9803	Peru	White
26	CHEN189	D9400	Peru	White	56	CHEN364	D9855	Chile	Whitish-yellow
27	CHEN191	D9402	Chile	Bright	57	CHEN371	D9862	Chile	Yellow
28	CHEN193	D9404	Chile	White	58	CHEN390	D9878	Peru	Bright-white
29	CHEN194	D9405	Chile	Bright	59	CHEN391	D9879	Chile	White
30	CHEN195	D9406	Peru	Whitish-yellow	60	CHEN392	D9880	Peru	White

### DNA extraction and genotypic assessment

Genomic DNA was extracted from the young and fresh leaves at 2–4 leaf stage (25 days old) according to the CTAB method^25^. The quality and quantity of the extracted DNA were evaluated by electrophoresis on a 1% agarose gel and spectrophotometer (LAMBDA 1050UV). A total of 40 microsatellite (SSR) markers were used for molecular analysis and amplification of quinoa genomic DNA. The quality and quantity of extracted DNA on 1% agarose gel and spectrophotometric method were determined. A total of 40 SSR markers were selected using previous studies^19,26^ (Table 2).

**Table 2:** Microsatellite (SSR) markers used for molecular evaluation of quinoa genotypes in this study.

Marker name	Forward sequence	Reverse sequence	Annealing temperature (°)	Expected PCR product size
KAAT001	tggctatatcatatgcgtaatgtg	gggctcagattgtatctcgac	59	176
KAAT006	tctgcaggatcggtaacctt	ttgtatctcggcttcccact	54	171
KAAT007	aggtacaggcgcaaggatac	cggtagcatagcacagaacg	55	197
KAAT023	agattgtatctcggctttcca	cacttcattgtattgcatttagga	51	225
KAAT024	cctaatgccacggtttccta	ccgctgaatagacacccagt	57	199
KAAT025	gagtgggagcccagattgta	agcaaagtaaatttcaacaaagca	53	160
KAAT026	cggagtcagatggttctggt	tcaagtgcagctcaatcacc	60	179
KAAT027	tttaaactttattgacccttggaaa	ggatgctattgcattgctga	57	192
KAAT030	tcaaatatgtgtggaccactctaag	ccaatttcttgtaaattgattgactt	53	203
KAAT031	agagaccaatgccggataga	gttcgctatagctagaggagtgg	51	205
KAAT033	tgccaactgacgagacaaag	gcgggagctcatatcttcac	62	208
KAAT036	ggcagcgatcgtgaaata	gggacccaaattgtatctcg	63	254
KAAT040	gcatgagtggtaatggagga	cttgaaggagcagtattattcac	67	166
KAAT047	tctcggttccctactaatttcttg	tttatgcagcaagggttgtaaa	51	196
KAAT050	tcatgcctaggatcttgcttt	tcgtatacggactaaattgtccac	57	158
KCAA006	ttgagcaggatgatgtggag	ttggagaaacataccttgttgg	55	165
KCAA014	gaatttgcatgcccttcatt	ccgccctcgctactatgat	53	176
KCAA015	tggttggaggcaaacatacc	tgagggtgaagaggaggatg	58	204
KCAA019	gtagttgggcggatgtgtct	gcgactgagctagcaggttt	56	163
KCAA022	ccaattgcatgctcctcatt	aatgcaaacatgggaggaga	60	153
KCAA106	atatggaagtcggccaacag	gcatgctcatcatttgttgc	63	148
KCAA107	caccagaaccctcgatctaca	tggttactgttgttgttgttaatttg	67	264
KGA002	aaagaacgcatccttccaat	aacctagccaacactccctaaa	56	198
KGA003	attgccgacaatgaacgaat	atgtaaatggcatgtcccaac	61	158
KGA006	aaacaaattctatcattcggttagg	gccaacgagcctgatgtaa	66	168
KGA041	tttggtgcaaatgttgttca	ttccaagaccaaaccctctc	57	234
KGA042	ttggtagtgggtaagagaacctg	ctccctccagccacataatc	51	193
KGA047	gcagtgcatgaatttggaca	gaagctggcaccttatacatgc	55	179
KGA048	acgtcgaggatggctaggt	ccaacaatcatcatcaataccc	58	207
KGA053	aaatttctgcctctgtgcaa	ctcaaacttctgcctcctga	53	186
KGA054	tgttgattgataatatgtaatggtgga	cattcataacagcgagagatgg	58	199
KGA055	cccaacccaccaaacttaca	gaaaggaaagtgattgcaaagaa	56	181
KGA056	gactaacggtgtccaaactgc	ccttctgcattacaccgtca	60	190
KGA059	ataaccactccgatggcaaa	cagccacctggcagttaga	63	171
KGA109	accttgaaccacaccgaaac	tcgctgctcatcaccatatt	51	161
KGA111	aatggtaaacagaccagactagca	tgggttcatttagtagaatcaagg	57	149
KGA114	tgttgagtgcgctttaatgg	aataggtgtagccgcgtagg	53	178
KGA116	ccttccttctctacgctctcc	tgggacccaaatctttcatag	62	173
KGA117	gctttgtagacacctgtcatgg	ccactccgatgataaagttagaatg	63	193
KGA118	gctgtgtttgacccatgttg	caaccacagcaaaggtgtga	67	183

The amplification (PCR) reaction was carried out in a reaction solution of 20 μl containing 2 μl of PCR buffer (10×), 0.96 μl of MgCl2 (50 mM), 1.2 μl of dNTPs (2 mM), 0.8 μl of forward and reverse primers each of 10 pmol concentration, 1.2 μl of Taq DNA Polymerase (1 U/μl), 4 μl of the template DNA (70 ng/μl), and 9.06 μl of ddH_2_O^27^. The PCR amplification was carried out in a thermal cycler (PTC 100, M.J. Research, Inc.) at the Genomics Laboratory of the Faculty of Agricultural Sciences, University of Guilan, Iran. The thermal cycle protocol included initial denaturation at 94 °C for 5 min, followed by 30 cycles of 94 °C for 1 min (denaturation), 55°C for 1 min (annealing), 72 °C for 1 min (extension), and final extension at 72 °C for 5 min^27^. A total of 3 μl PCR products were denatured and separated by vertical electrophoresis system on 6% polyacrylamide denaturing gels, and electrophoretic bands were revealed using silver staining^28^.

### Data analysis

For phenotypic data, descriptive statistics including minimum, maximum, mean, standard deviation, and coefficient of variation were calculated. The molecular data were manually scored using the binary coding system, (1) for presence and (0) for absence of each specific allele in all studied genotypes according to molecular weight using DNA size marker 100 bp Fermentas. The number of observed and polymorph alleles for each SSR locus in all quinoa genotypes was calculated. The effective number of alleles, gene diversity index and Shanon’s diversity index were evaluated as described by Kimura and Crow^29^, Nei^30^ and Lewontin^31^ respectively. The polymorphic information content (PIC) index, indicating the ability of each marker to distinguish the genotypes^32^, was also calculated as expected heterozygosity based on the studies of Botstein et al.^33^. All genetic diversity indices were calculated using the PopGene software ver. 1.32^34^, and the Power Marker software ver. 3.25^35^. Cluster analysis using the neighbor-joining method was used to group and determine the differences and/or similarities between genotypes. Also, principal coordinates analysis (PCoA) used to display the two-dimensional distribution of quinoa genotypes using the studied microsatellite markers.

To perform association study, the effective population structure of the studied genotypes into separate subpopulations and identification of mixed genotypes was first performed using the Bayesian method by Structure software^36^. This method attributes each of the genotypes to a hypothetical subpopulation with a probability level, so that the degree of linkage disequilibrium in each subpopulation is minimum and the gametic stage equilibrium is maximum. The initial hypothetical subpopulation values were considered from 1 to 10 and to increase the accuracy, ten replications were performed for each subpopulation. Therefore, the admixture model and the allelic frequency independence with 10,000 burn-in and 10,000 Markov Chain Monte Carlo (MCMC) models, were used to obtain the maximum likelihood curve^37^. Structure software calculates a Qst matrix for each K value (actual number of subpopulations), which includes estimating the probability coefficients of membership of each genotype in each subpopulation. In the resulting plot, when the membership percentage of a genotype to a cluster is greater than or equal to 0.7, the genotype is assigned to that cluster, but if the membership percentage is less than 0.7, it is defined as a mixed genotype^38^. The actual number of subpopulations (K) was determined based on ΔK statistic using Evano et al.^39^ method.

General linear model (GLM) and mixed linear model (MLM) were used to identify informative and linked markers with the evaluated traits using TASSEL 3.0 software^40^. In the general linear model, the population structure coefficient matrix (Q matrix) is only used to determine the relationship between markers and traits, while in the mixed linear model, kinship relationships matrix (K matrix) along with Q matrix (K + Q matrices) are used to avoid false correlations between markers-traits^2^.

### Plant ethics statement

In this research, experimental research/field studies/collection of plant/plant material, complied with the relevant institutional, national, and international guidelines and legislation.

## Results and discussion

### Descriptive statistics

Descriptive statistics of the studied traits in 60 quinoa genotypes are presented in Table 3. The results showed that most of the studied traits had a relatively high diversity. The highest diversity was observed in grain saponin and grain protein with 18.54% and 16.24%, respectively. Since in association study, the relationship between phenotypic diversity of the studied traits with polymorphisms in the genome (genotypic diversity) are assessed and markers related to the target traits are identified, the mapping results will be more accurate by increasing the diversity of the studied traits. This diversity is the result of gene recombinations throughout the evolutionary history in natural populations, and in fact, all meiotic events during the evolutionary history of the organism are considered in association study^41^. Therefore, the existence of high diversity in the studied population in this research can provide more reliable and credible results for association studies.

**Table 3:** Descriptive statistics of the studied traits in 60 quinoa genotypes.

Trait	Minimum	Maximum	Range	Mean ± SD	CV (%)
Days to flowering	65.28	103.23	38.05	83.76 ± 1.78	8.34
Days to maturity	105.31	148.23	42.92	131.32 ± 3.24	11.21
Plant height (cm)	77.34	132.16	54.82	81.76 ± 8.25	10.27
Panicle length (cm)	12.24	38.16	25.92	29.3 ± 0.26	7.64
Grain length (mm)	1.43	2.58	1.15	1.75 ± 0.09	12.76
Grain saponin (%)	0.07	1.91	1.84	0.97 ± 0.08	18.54
Grain protein (%)	10.21	23.34	13.13	18.22 ± 0.57	16.24
1000-grain weight (g)	1.55	3.96	2.41	2.43 ± 0.45	13.24
Grain yield (kg ha^-1^)	2166.3	4208.3	2041.7	3285.44 ± 2.06	15.39
Harvest index	28.19	58.23	30.04	34 ± 2.06	11.29

### Correlation coefficients

The results of phenotypic and genotypic correlation coefficients (Table 4) among the studied traits showed that the highest positive and significant phenotypic and genotypic correlations were observed between grain yield and harvest index (0.99, 0.98), 1000-grain weight (0.98, 0.92), grain protein (0.91, 0.86), grain length (0.89, 0.78), and panicle length (0.86, 0.75) (Table 4). Also, the phenotypic and genotypic correlations between harvest index with 1000-grain weight, grain length, grain protein and panicle length, as well as between grain protein with panicle length and grain length were positive and highly significant (Table 4). The traits with high correlations with grain yield can be used as indirect selection criteria to improve grain yield in future breeding programs.

**Table 4:** Phenotypic (lower diagonal) and genotypic (upper diagonal) correlation coefficients among the studied traits in the 60 quinoa genotypes.

Row	Trait	1	2	3	4	5	6	7	8	9	10
1	Days to flowering	1	− 0.12	0.19	0.16	0.14	− 0.21	0.10	0.05	0.05	0.07
2	Days to maturity	− 0.18	1	− 0.22	0.33	0.18	0.08	0.21	0.23	0.25	0.24
3	Plant height (cm)	0.25	− 0.25	1	− 0.08	− 0.14	0.08	0.41	− 0.07	− 0.14	0.09
4	Panicle length (cm)	0.21	0.37	− 0.08	1	0.76	− 0.14	0.79	− 0.42	0.75	0.79
5	Grain length (mm)	0.10	0.25	− 0.19	0.79	1	0.01	0.76	0.66	0.78	0.83
6	Grain saponin (%)	− 0.28	0.03	0.08	− 0.14	0.01	1	− 0.10	− 0.09	− 0.09	− 0.07
7	Grain protein (%)	0.14	0.24	0.35	0.84	0.84	− 0.11	1	0.78	0.86	0.80
8	1000-grain weight (g)	0.00	0.28	− 0.11	− 0.34	0.79	− 0.13	0.87	1	0.92	0.91
9	Grain yield (kg ha^−1^)	0.01	0.30	− 0.18	0.86	0.89	− 0.10	0.91	0.98	1	0.98
10	Harvest index	0.02	0.29	− 0.14	0.88	0.90	− 0.09	0.89	0.96	0.99	1

The numbers higher than 0.32 are statistically significant.

Other researchers have reported similar results. Saddiq et al.^42^ found a positive and significant correlation between grain yield with panicle length and 1000-grain weight. Also, a positive and significant correlation between spike length and seed length by Manjarres-Hernández et al.^43^ and between grain yield and harvest index by Hussain et al.^44^ was previously reported, which was consistent with the results of the current study. In contrast, Reguera et al.^45^, Hussain et al.^44^, Granado-Rodríguez et al.^46^, and Matías et al.^47^ reported a negative and significant correlation between grain yield and grain protein, which was not consistent with the results of this study. Different results in different studies can be obtained due to the use of different genetic materials as well as different environmental conditions of the experiment.

### Genetic diversity of the studied population based on SSR markers

The genetic diversity indices of SSR markers in the sixty studied quinoa genotypes are shown in Table 5. A total of 140 scorable alleles with an average of 3.5 alleles were amplified by 40 SSR markers, of which 136 alleles (97%) were polymorph. Among the studied markers, KAAT027, KAAT036, KAAT040, KCAA014, KGA042, KGA055 and KGA059 with five alleles showed the highest number of polymorph alleles. The effective number of alleles varied from 1.08 (marker KAAT006) to 3.72 and 3.75 (markers KAAT036 and KAAT027, respectively), with an average of 1.81 alleles. The polymorphic information content (PIC) ranged from 0.23 (marker KAAT007) to 0.89 (marker KAAT023) with an average of 0.60. The average values of Shannon’s diversity index and Nei’s gene diversity index were 0.54 and 0.44 per each SSR locus, respectively. Marker KAAT027 indicated the highest values for both Shannon’s (0.82) and Nei’s gene (0.80) diversity indices and marker KAAT007 showed the lowest values (0.15 and 0.09, respectively) (Table 5). In total, relatively high diversity was observed for most of the studied markers, which indicates the appropriate selection and high efficiency of the selected markers in this study. Therefore, markers KAAT023, KAAT027, KAAT036, and KCAA014 showed the highest values for most of the diversity indices in this research and can be recommended as the highest appropriate markers to evaluate genetic diversity in quinoa. On the other hand, it seems that this marker type is the most informative for assessing genetic diversity in quinoa and for discriminating among the varieties.

**Table 5:** Diversity indices calculated for the studied SSR markers in 60 quinoa genotypes.

SSR marker	Number of observed alleles	Number of polymorph alleles	Effective number of alleles	Polymorphic information content	Shannon’s diversity index	Nei’s gene diversity index
KAAT001	4	4	2.16	0.56	0.58	0.45
KAAT006	2	2	1.08	0.32	0.21	0.14
KAAT007	2	2	1.15	0.23	0.15	0.09
KAAT023	3	3	2.33	0.89	0.74	0.70
KAAT024	3	3	1.62	0.76	0.63	0.58
KAAT025	3	3	2.12	0.72	0.69	0.65
KAAT026	4	4	2.35	0.46	0.35	0.29
KAAT027	5	5	3.75	0.85	0.82	0.80
KAAT030	2	2	1.11	0.81	0.65	0.59
KAAT031	3	3	1.59	0.76	0.61	0.57
KAAT033	2	2	1.13	0.73	0.65	0.60
KAAT036	5	5	3.72	0.85	0.78	0.75
KAAT040	5	4	3.13	0.77	0.63	0.59
KAAT047	4	4	2.33	0.43	0.35	0.29
KAAT050	2	2	1.13	0.79	0.69	0.62
KCAA006	2	2	1.18	0.85	0.74	0.68
KCAA014	5	5	2.64	0.88	0.79	0.74
KCAA015	2	2	1.24	0.83	0.67	0.63
KCAA019	2	2	1.12	0.79	0.69	0.65
KCAA022	3	3	1.38	0.59	0.46	0.32
KCAA106	4	4	1.61	0.48	0.38	0.35
KCAA107	3	3	1.73	0.58	0.54	0.46
KGA002	3	2	1.23	0.49	0.40	0.28
KGA003	4	4	1.51	0.56	0.43	0.37
KGA006	3	3	1.29	0.64	0.35	0.28
KGA041	4	4	2.16	0.62	0.55	0.46
KGA042	5	5	1.95	0.33	0.63	0.41
KGA047	4	4	1.32	0.48	0.37	0.27
KGA048	3	3	1.71	0.44	0.43	0.36
KGA053	4	4	1.81	0.59	0.59	0.51
KGA054	4	3	1.64	0.61	0.59	0.53
KGA055	5	5	2.35	0.68	0.71	0.55
KGA056	3	3	1.69	0.39	0.33	0.30
KGA059	5	5	1.61	0.51	0.57	0.42
KGA109	4	4	1.51	0.55	0.36	0.23
KGA111	3	2	1.33	0.44	0.47	0.37
KGA114	5	5	2.48	0.61	0.65	0.51
KGA116	3	3	1.57	0.46	0.48	0.38
KGA117	4	4	1.55	0.54	0.46	0.38
KGA118	4	4	1.39	0.39	0.44	0.35
Mean	3.5	3.4	1.81	0.60	0.54	0.44

Several studies had previously reported the high PIC values and polymorphism for microsatellite markers in quinoa populations^1,19,26,48^. Maughan et al.^11^ used microsatellite markers to study quinoa germplasm to introduce highly reproducible and informative microsatellite markers. A total of 1276 gene loci with ATG, ATT, and CA repeats in the genomic library were sequenced, and the microsatellite markers were designed for 397 gene loci. The number of observed alleles in each gene locus was between 2 and 13 alleles with an average of 4 alleles in each locus, and the heterozygosity of markers was 57% on average. They observed high heterozygosity in 67 markers and introduced these markers as appropriate for linkage mapping as well as use in quinoa breeding programs^11^. Jarvis et al.^19^ also used 216 SSR markers to construct a quinoa linkage map. The average heterozygosity value of these markers was calculated to be 0.57. They constructed the first linkage map of quinoa with 216 SSR markers in a recombinant inbred lines (RILs) population consisting of 38 linkage groups with a total length of 913 cM^19^.

### Population structure

**Figure 1:**
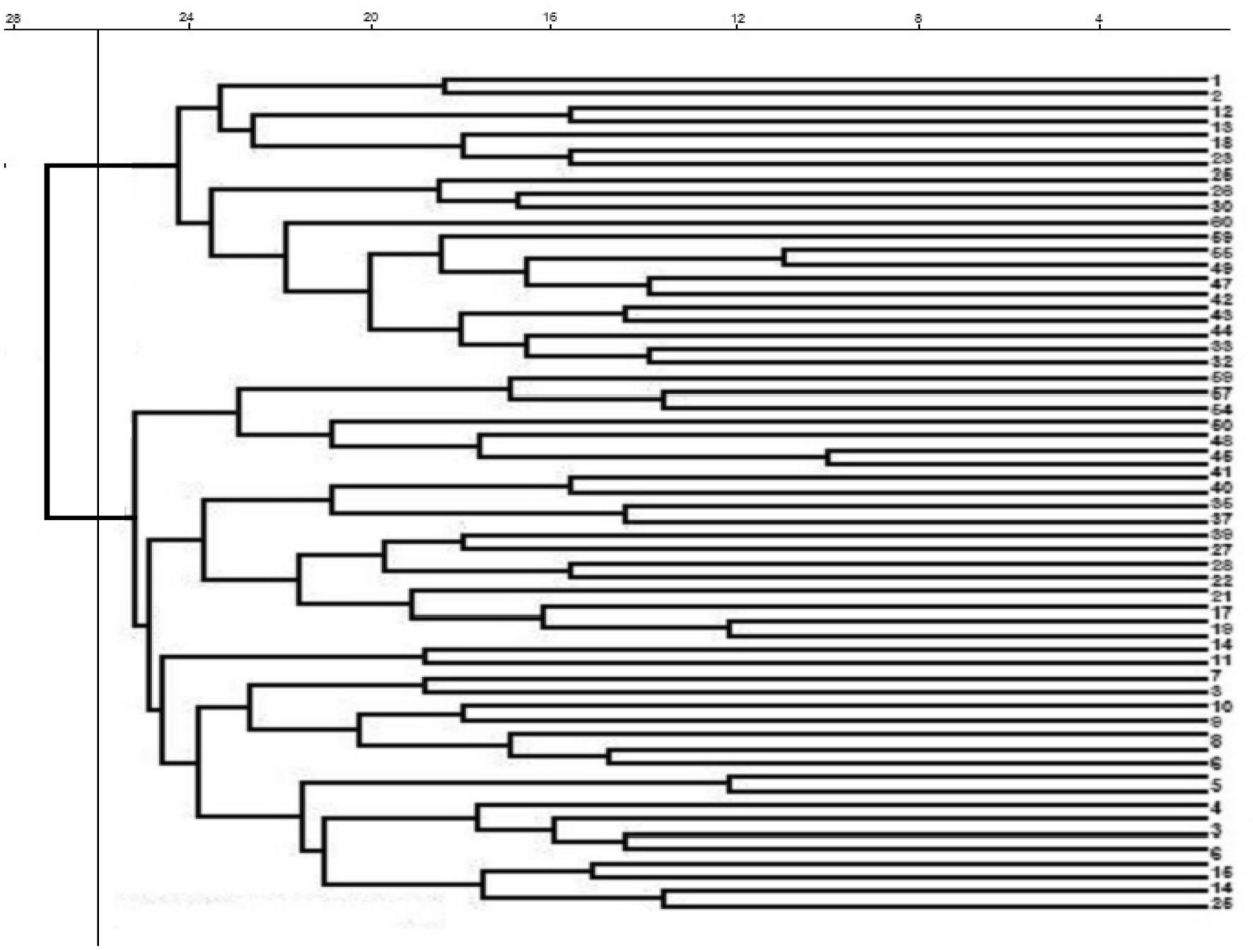
Dendrogram of cluster analysis for grouping 60 quinoa genotypes using 40 SSR markers.

**Figure 2:**
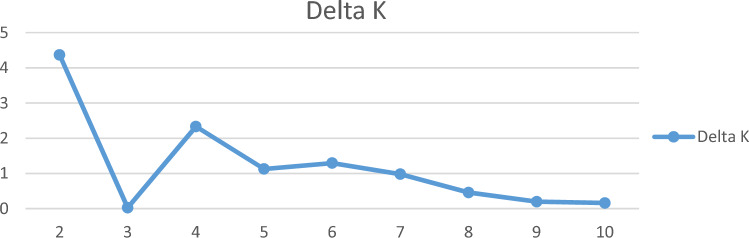
Bilateral charts to determine the number of sub-populations in the studied quinoa genotypes (K = 2) based on microsatellite markers.

**Figure 3:**
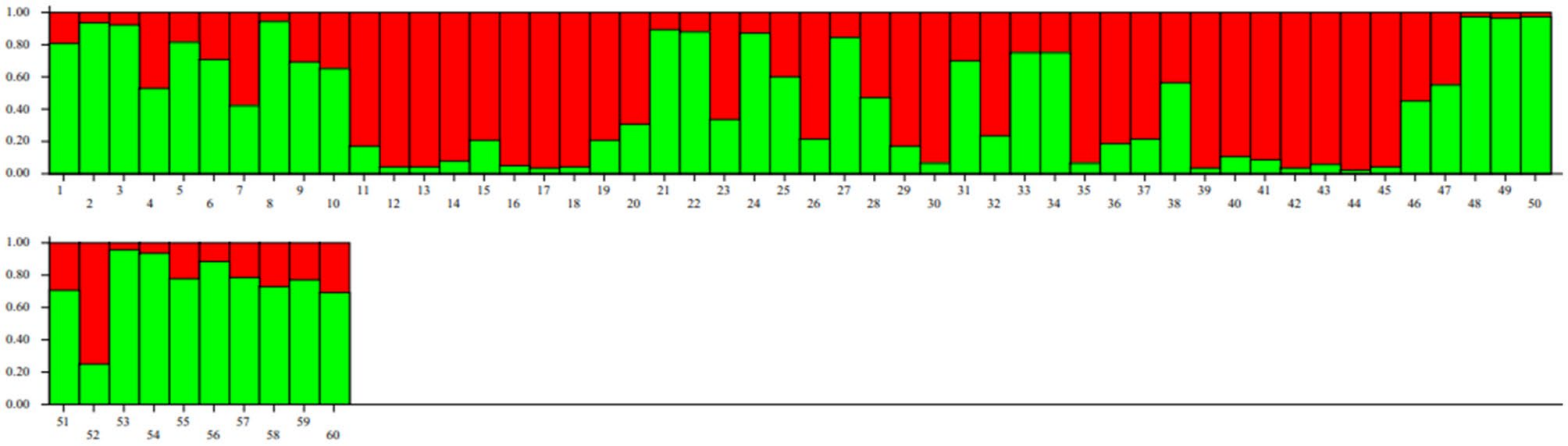
Barplot of structure analysis of the 60 studied quinoa genotypes based on 40 microsatellite markers using Bayesian model. Each color indicates one subpopulation or cluster (K = 2). The vertical axis shows the membership coefficient of each genotype into each cluster.

In the genetic studies, population structure which shows the relationships of individuals inter- and intra-population, provides a perspective on the evolutionary relationships between individuals in a population. In an ideal association study, the studied population should not have a specific structure and not be structurally divided into different subpopulations. If the effect of population structure and kinship relationships on association studies is not considered, false positive results will be obtained^49,50^. To identify the population structure in this research, the studied quinoa genotypes were first grouped by cluster analysis using Neighbor-Joining method (Fig. 1). The results showed that 60 quinoa genotypes could be divided into two subpopulations with 38 and 22 genotypes, respectively. This grouping did not correspond to origin or seed color, so that genotypes with different origin or seed color were grouped in each subpopulation. Grouping of genotypes with different origins and seed colors only in two subpopulations can probably be due to the exchange of genetic materials and/or the mixing of different seeds, as well as the existence of two different types, highland and lowland, among the quinoa genotypes studied in this research. Patiranage et al.^32^ using whole-genome sequencing of 310 quinoa accessions, clustered the quinoa accessions into two main groups, highland and lowland. Furthermore, to analyze the population structure and determine the actual number of subpopulations, the STRUCTURE software ver. 4.0 was used and the optimal number of subpopulations was calculated based on the change of ΔK versus K values. The results indicated that the number of possible subpopulations in the studied quinoa genotypes is equal to two subpopulations (Fig. 2). Therefore, K = 2 was considered as the optimal number of subpopulations to analyze the population structure and calculate the membership coefficient of individuals in each cluster (Q-matrix). As shown in Fig. 3, among 60 genotypes studied in the current research, 29 (48%) and 23 (38%) genotypes belonged to the first and second subpopulations, respectively, and eight genotypes (13%) had a membership coefficient of less than 0.7 and were considered mixed genotypes. El-Harty et al.^21^ studied the population structure of 32 quinoa genotypes using STRUCTURE software and identified two possible clusters (K = 2), with 13 genotypes (41%) belonging to the first cluster and 8 genotypes (25%) belonging to the second cluster, and 11 genotypes (34%) also having mixed structure.

**Figure 4:**
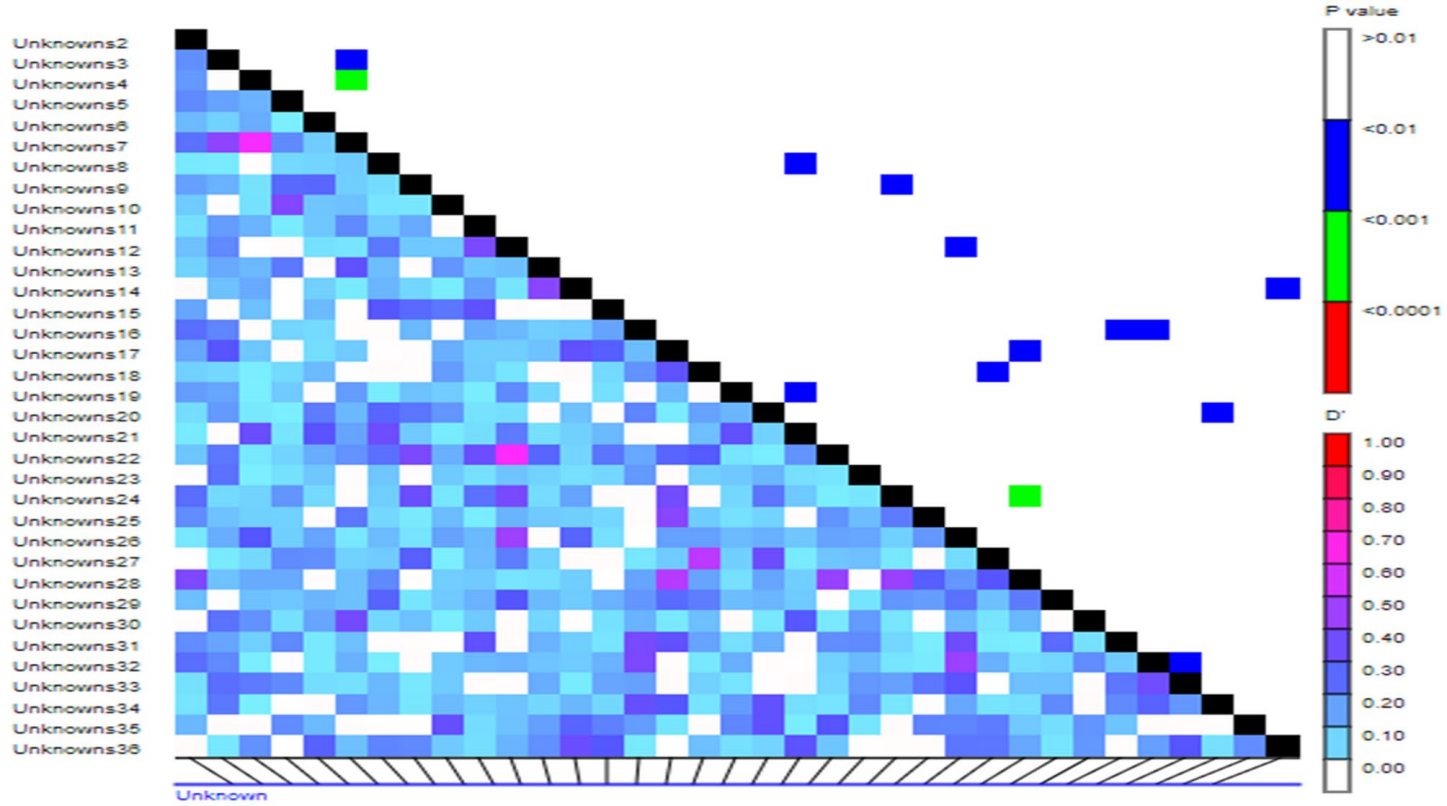
Linkage disequilibrium (LD) plot. The upper and lower diagonal represent linkage disequilibrium and p-value statistics for each marker pair, respectively.

In addition to the population structure, the linkage disequilibrium (LD) is another factor affecting association study. Coinheritance of two or more loci on a chromosome within a genetic region is called genetic linkage^27^. Linkage equilibrium is the random combinations of alleles at various loci where observed haplotype frequencies agree with the predicted haplotype frequencies in a population, but LD is a non-random association of alleles at various loci and describes non-equal haplotype frequencies^51^. Linkage disequilibrium values between the studied marker pairs in the present study along with their significant probability levels are shown in Fig. 3. Several demographic and genetic factors are affecting LD significantly, out of which mutation and recombination are the key factors^52^. Population structure, new mutations, autogamy, genetic drift, epistasis, genomic rearrangement, selection and kinship significantly increase LD, while higher recombination and mutation rates, repetitive mutations, gene conversion and outcrossing significantly decrease LD^53^. Nevertheless, significant LD is often observed between distant loci located even on different chromosomes causing spurious associations in association studies. It should be noted that the selection of a population with higher or lower LD levels depends on the objective of the mapping study^27^. The extensive level of LD (long-stretched or genetically unlinked LD) reduces the number of markers required for marker-trait association (MTAs) but lowers the mapping resolution, whereas less extensive or short-stretched LD needs a relatively larger number of markers to identify a gene but increases mapping resolution^51^ (Fig. 4).

### Principal coordinates analysis (PCoA)

**Figure 5:**
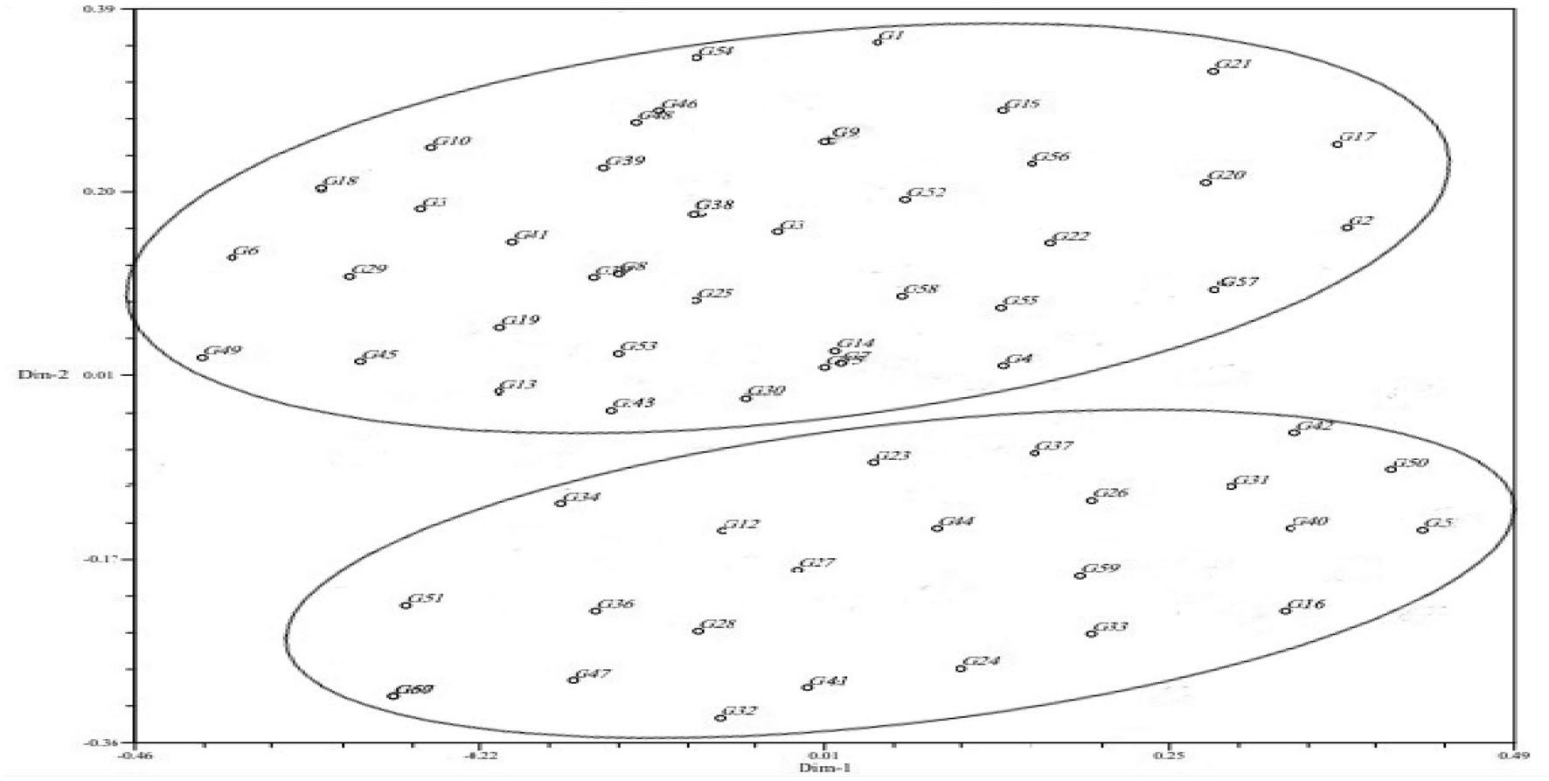
Two-dimensional plot of principal coordinate analysis based on the first two components in 60 quinoa genotypes.

Principal coordinates analysis (PCoA) can be used to display a two-dimensional distribution of people (and/or genotypes and populations) based on genetic markers. The gathering of people in one area of the plot shows their genetic similarity^54,55^. The results of PCoA of the studied quinoa genotypes using microsatellite markers for the is presented in Table 6. The results showed that the first and second components explained 9.78% and 7.63%, and the first ten components accounted for 61.20% of the total variance of the studied population. In studying genetic diversity using molecular data, the markers should have a uniform and appropriate distribution on the genome level, so that they can cover the entire genome well^56,57^. The justification of lower values of the total variance by the first few components in this study indicates the appropriate distribution and optimal sampling of the markers used in the whole genome. The diagram of the two-dimensional distribution of the quinoa genotypes based on the first and second principals resulting from the PCoA is presented in Fig. 5. As shown in Fig. 5, 60 quinoa genotypes were divided into two separate groups, including 22 and 38 genotypes, respectively. The result of PCoA confirmed the number of groups derived from the cluster analysis, although the genotypes within the groups were not the same in these two analyses. This difference is somewhat natural and obvious, because the cluster analysis grouped the genotypes based on 100% of the information obtained from the markers, while the grouping of the genotypes in the PCoA was done based on the first and second components, which explained 17.42% of the total variance (Table 6).

**Table 6:** Variance percentage explained by first ten components of the principal coordinate analysis in 60 quinoa genotypes.

Principal component	Variance explained by each component	Cumulative variance percentage
1	9.78	9.78
2	7.63	17.42
3	7.15	24.58
4	6.81	31.39
5	6.22	37.62
6	5.56	43.17
7	5.23	48.40
8	4.69	53.09
9	4.27	57.35
10	3.85	61.20

### Association study using GLM and MLM models

The main challenge in association study is to identify real and false relationships between the studied markers and traits according to the population structure and kinship relationships, although the influence of kinship relationships in providing false positive results is more than the population structure^58–60^. To compare the identified markers between the first and second years, as well as to detect the stable and true markers associated with traits in both experimental years to use them in breeding programs, an association study using GLM and MLM models was separately done for data of the first and second years.

The results of the association study in the first year (2019) showed 35 and 32 significant marker-trait associations (MTAs) using the GLM and MLM models, respectively (Table 7). Grain yield traits with five MTA and days to flowering and grain saponin with two MTA had the highest and lowest significant associations with the markers used in this research, respectively. Coefficient of determination (R^2^) statistics shows the percentage of the phenotypic variance explained by each marker (Table 7). R^2^ values exhibited by the investigated markers in the first year (2019) varied from 0.11 to 0.33 in the GLM model and from 0.07 to 0.30 in the MLM model. The highest and lowest R^2^ values in the GLM model belonged to the KAAT001 and KGA114 markers associated with grain yield and grain saponin, respectively, and in the MLM model belonged to the KGA002 and KGA111 markers both associated with panicle length (Table 7). Therefore, two markers KAAT001 and KGA002 are useful and informative markers to identify the genes encoding respective traits. These markers can be used as reliable markers to improve target traits using marker-assisted selection methods.

**Table 7:** SSR markers linked to measured traits in the studied quinoa genotypes based on GLM and MLM models in both years.

Trait	2019					2020				
	SSR marker	GLM		MLM		SSR marker	GLM		MLM	
		p-value	R^2^	p-value	R^2^		p-value	R^2^	p-value	R^2^
Days to flowering	KAAT031	0.03	0.18	0.02	0.16	KAAT001	0.01	0.20	0.04	0.15
	KAAT025	0.08	0.22	0.02	0.18	KAAT025	0.02	0.18	0.06	0.13
	KAAT007	0.01	0.26	0.01	0.19	KAAT031	0.02	0.16	0.03	0.15
Days to maturity	KAAT040	0.01	0.25	0.02	0.21	KAAT024	0.01	0.27	0.01	0.23
	KAAT027	0.05	0.15	0.01	0.25	KAAT023	0.04	0.16	0.01	0.27
	KAAT030	0.03	0.18	0.07	0.14	KAAT030	0.05	0.14	0.03	0.21
Plant height (cm)	KCAA106	0.03	0.18	0.06	0.17	KAAT033	0.02	0.19	0.07	0.11
	KGA117	0.01	0.24	0.06	0.16	KGA117	0.06	0.20	0.02	0.16
	KGA111	0.07	0.21	0.03	0.22	KGA042	0.07	0.18	0.01	0.20
	KAAT030	0.02	0.20	0.01	0.28	KAAT030	0.04	0.16	0.05	0.10
Panicle length (cm)	KCAA022	0.04	0.17	0.01	0.26	KGA109	0.03	0.19	0.06	0.09
	KGA002	0.01	0.22	0.01	0.30	KGA003	0.01	0.25	0.01	0.22
	KGA003	0.02	0.18	0.03	0.16	KCAA019	0.03	0.20	0.04	0.17
	KGA111	0.05	0.12	0.08	0.07	KGA047	0.01	0.27	0.04	0.16
Grain length (mm)	KCAA107	0.02	0.20	0.01	0.25	KGA003	0.01	0.24	0.03	0.18
	KGA006	0.01	0.16	0.09	0.13	KCAA106	0.03	0.18	0.02	0.20
	KCAA015	0.01	0.25	0.02	0.22	KCAA022	0.01	0.26	0.08	0.09
	KCAA022	0.03	0.18	0.03	0.19	KAAT033	0.08	0.09	0.02	0.23
Grain saponin (%)	KGA118	0.03	0.18	0.05	0.16	KGA041	0.03	0.19	0.02	0.22
	KGA114	0.07	0.11	0.01	0.19	KAAT026	0.04	0.16	0.09	0.06
	KGA041	0.01	0.26	0.08	0.09	KAAT027	0.02	0.23	0.02	0.24
	KGA003	0.09	0.20	0.02	0.26	KGA117	0.01	0.25	0.01	0.27
Grain protein (%)	KGA109	0.01	0.27	0.01	0.26	KCAA022	0.01	0.28	0.01	0.25
	KGA059	0.02	0.23	0.03	0.21	KGA059	0.03	0.20	0.03	0.21
	KGA116	0.03	0.18	0.04	0.16	KGA048	0.02	0.25	0.02	0.20
1000-grain weight (g)	KCAA107	0.02	0.23	0.01	0.26	KAAT026	0.02	0.22	0.01	0.16
	KAAT036	0.01	0.30	0.01	0.22	KGA116	0.01	0.28	0.02	0.23
	KGA002	0.01	0.28	0.02	0.25	KCAA015	0.02	0.29	0.05	0.28
	KAAT023	0.02	0.21	0.05	0.19	KGA002	0.03	0.20	0.01	0.26
	KAAT006	0.05	0.14	0.07	0.13	KAAT023	0.01	0.25	0.01	0.23
Grain yield (kg ha^−1^)	KAAT001	0.01	0.33	0.01	0.28	KGA003	0.01	0.27	0.02	0.21
	KAAT023	0.01	0.28	0.01	0.26	KGA053	0.01	0.26	0.02	0.20
	KGA003	0.02	0.27	0.05	0.12	KAAT023	0.03	0.25	0.03	0.17
	KAAT047	0.03	0.21	0.03	0.14	KGA111	0.02	0.27	0.03	0.15
	KGA006	0.04	0.17	0.03	0.16	KGA002	0.03	0.19	0.04	0.13
	KGA002	0.05	0.15	0.02	0.19	KAAT040	0.04	0.16	0.01	0.27
Harvest index	KGA055	0.01	0.20	0.01	0.22	KGA118	0.03	0.18	0.01	0.23
	KAAT040	0.02	0.17	0.03	0.18	KAAT040	0.01	0.23	0.03	0.24
	KGA003	0.06	0.12	0.05	0.15	KGA055	0.05	0.11	0.01	0.20
	KGA118	0.01	0.19	0.09	0.11	KCAA014	0.01	0.25	0.05	0.22

The results of the association study in the second year (2020) showed 37 and 35 significant marker-trait associations (MTAs) using the GLM and MLM models, respectively (Table 7). Grain yield traits with six MTA and plant height with two MTA had the highest and lowest significant associations with the markers used in this research, respectively. Coefficient of determination (R^2^) statistics shows the percentage of the phenotypic variance explained by each marker (Table 7). R^2^ values exhibited by the investigated markers in the second year (2020) varied from 0.09 to 0.29 in the GLM model and from 0.06 to 0.28 in the MLM model. The highest and lowest R^2^ values in the GLM model belonged to the KCAA015 and KAAT033 markers associated with grain yield and Grain length, respectively, and in the MLM model belonged to the KCAA015 and KAAT026 markers associated with 1000-grain weight and grain saponin (Table 7). Therefore, marker KCAA015 is a useful and informative marker to identify the genes encoding respective traits. These markers can be used as reliable markers to improve target traits using marker-assisted selection methods^61^.

The results showed that some significant MTAs in the first year (2019) were only identified using the GLM model, such as the association of KAAT030 with days to maturity, KCAA106 and KGA117 with plant height, KGA006 with grain length, KGA041 with grain saponin, KAAT006 with grain yield, and KGA118 with harvest index, and some others only using the MLM model, such as KAAT025 with days to flowering, KGA111 with plant height, KGA114 and KGA003 with grain saponin, and KAAT047 with grain yield. Also, in the second year (2020), some significant MTAs were only identified using the GLM model, such as KAAT025 with days to flowering, KGA109 with panicle length, KCAA022 with grain length, and KAAT026 with grain saponin, and some others only using the MLM model, such as KGA117 with plant height, and KAAT033 with grain length. These results indicated that the difference in the number and type of the identified and associated markers with the studied traits depends on the type of input data and the type of statistical model used to identify significant marker-trait associations.

However, some significant MTAs were commonly identified by both GLM and MLM models (Table 8). Also, many markers identified by both GLM and MLM models had a significant association with more than one trait, among which the association of KAAT030 and KGA111 with plant height and the relationship of KGA003, KGA006 and KAAT023 with grain yield in the first year (2019) and association of KGA117 and KAAT026 with Grain saponin and the relationship of KAAT040, KGA002 and KAAT023 with grain yield in the second year (2020) can be mentioned. This can be due to the pleiotropic effects of a gene or the effect of linkage between adjacent genes. Identifying markers that are simultaneously associated with several traits will be of great importance to breeders, as they can be used in the simultaneous improvement of multiple traits^22^. Furthermore, some markers identified in this study had a low R^2^ value, indicating the nature of quantitative and multigenic inheritance of the investigated traits.

**Table 8:** Significant MTAs identified by both GLM and MLM models in in 2019–2020 years and stable MTAs identified in both years.

Year	Marker	Trait
2019	KAAT030	Days to maturity, Plant height
	KGA111	Plant height, Panicle length
	KCAA022	Panicle length, Grain length
	KGA003	Grain length, Grain saponin, Grain yield, Harvest index
	KCAA107	Panicle length, 1000-grain weight
	KGA006	Grain length, Grain yield
	KAAT023	1000-grain weight, Grain yield
2020	KAAT030	Days to maturity, Plant height
	KGA117	Plant height, Grain saponin
	KCAA022	Grain protein, Grain length
	KAAT026	Grain saponin, 1000-grain weight
	KAAT040	Grain yield, Harvest index
	KGA002	1000-grain weight, Grain yield
	KAAT023	Days to maturity, 1000-grain weight, Grain yield
Stable MTAs identified in both years	KAAT030	Days to maturity, Plant height
	KAAT031	Days to flowering
	KGA117	Plant height
	KGA003	Panicle length, Grain length, Grain yield
	KGA041	Grain saponin
	KGA059	Grain protein
	KGA116, KGA002	1000-grain weight, Grain yield
	KAAT040	Grain yield, Harvest index
	KGA055	Harvest index

Based on the results of the association study in two experimental years, ten SSR markers linked to the evaluated traits were identified in both years (Table 8). Among which the association of KAAT030 and KGA117 with plant height and the relationship of KGA003, KGA116, KGA002 and KAAT040 with grain yield can be mentioned which can be used as the informative and stable markers for the genetic diversity and mapping studies in quinoa.

Mizuno et al.^62^ generated 136 quinoa inbred lines for the molecular identification and characterization of gene functions, and by using genotyping-by-sequencing analysis of the inbred lines, identified 5753 single nucleotide polymorphisms (SNPs) in the quinoa genome, and grouped the inbred lines into three genetic subpopulations. They measured physiological characteristics such as salt tolerance and key growth traits including 1000-grain weight, plant height, stem diameter, leaf dry weight, grain yield per plant, and days to flowering, and generated a heatmap that provided a brief overview of the genotype–phenotype relationship between quinoa inbred lines. They also performed an association study using 3156 SNPs and 12 phenotypic traits and identified 3091 and 4 significant MTAs based on GLM and MLM models, respectively. They indicated that the MLM model detected far fewer MTAs than the GLM model and suggested that the MLM may yield fewer false-positive results than the GLM because the MLM used the population structure and the kinship relationships^62^.

Patiranage et al.^32^ using whole-genome sequencing of 310 quinoa accessions, identified 2.9 million polymorphic high-confidence SNP loci and clustered the quinoa accessions into two main groups, highland and lowland, with F_ST_ divergence of 0.36 and fast LD decay of 6.5 and 49.8 kb, respectively. They also investigated the relationship between SNP markers and 17 agronomic traits using a genome-wide association study method and identified two candidate genes associated with thousand seed weights and a resistance gene analog associated with downy mildew resistance. They identified pleiotropically acting loci for four agronomic traits that highly responded to photoperiod, hence important for the adaptation to different environments.

## Conclusion

In this study, the genetic diversity of quinoa accessions was assessed using some important morpho-phenological characteristics including yield and yield components as well as microsatellite markers. Furthermore, the population structure and the possible number of subpopulations as well as the significant marker-trait associations were identified. The results showed that most of the studied traits had a relatively high diversity. A total of 140 scorable alleles with an average of 3.5 alleles per marker were amplified by 40 SSR markers, of which 136 alleles (97%) were polymorph. The relatively high diversity was also observed for most of the studied markers, but the markers KAAT023, KAAT027, KAAT036, and KGA006 showed the highest values for most of the diversity indices in this research and can be recommended as the appropriate and informative markers to evaluate the genetic diversity of quinoa.

The analysis of population structure using cluster analysis with the neighbor-joining method as well as the determination of the actual number of subpopulations by ΔK statistics using STRUCTURE software grouped the studied quinoa accessions into two possible subpopulations (K = 2). Out of the 60 genotypes studied in this research, 29 (48%) genotypes were allocated to the first and 23 (38%) to the second subpopulation, and eight genotypes (13%) were also considered as mixed genotypes.

Association studies using the GLM and MLM models identified the number of 35 and 32 significant MTAs for the first (2019) and 37 and 35 significant MTAs for the second (2020) experimental years, respectively. Among the significant MTAs identified for different traits, the highest number of significant MTAs were obtained for grain yield and 1000-grain weight with six and five MTAs, respectively. Also, some markers showed a significant relationship with more than one trait (including markers KAAT030 and KGA003 which are associated with days to maturity, plant height, grain length, grain saponin, grain yield, and harvest index), which can be due to the pleiotropic effects of a gene or the effect of linkage between adjacent genes. In total, based on the results of the association study in two experimental years, ten SSR markers linked to the evaluated traits were identified in both years, which can be used as the informative and stable markers for the genetic diversity and mapping studies in quinoa.

## Data Availability

The datasets used and/or analyzed during the current study are available from the corresponding author upon reasonable request.
